# Emerging insights into cuproptosis and copper metabolism: implications for age-related diseases and potential therapeutic strategies

**DOI:** 10.3389/fnagi.2024.1335122

**Published:** 2024-04-23

**Authors:** Haohui Fan, Kun Wang, Xiaofang Zhao, Bei Song, Tianci Yao, Ting Liu, Guangyu Gao, Weilin Lu, Chengyun Liu

**Affiliations:** Department of Geriatrics, Union Hospital, Tongji Medical College, Huazhong University of Science and Technology, Wuhan, China

**Keywords:** cuproptosis, age-related disease, copper, protein lipid acylation, mitochondria

## Abstract

The expanding geriatric population, whose predisposition toward disabling morbidities and age-related diseases (ARD) is well-documented, has become a paramount social issue, exerting an onerous burden on both the healthcare industry and wider society. ARD manifest as the progressive deterioration of bodily tissues and organs, eventually resulting in the failure of these vital components. At present, no efficacious measures exist to hinder the onset of ARD. Copper, an essential trace element, is involved in a wide range of physiological processes across different cell types. In recent research, a novel variant of copper-dependent cell death, termed cuproptosis, has been identified. This mode of cellular demise stands apart from previously recognized types of cell death. Cuproptosis occurs when copper binds with acyl-CoA synthetase in the tricarboxylic acid (TCA) cycle, resulting in protein aggregation and protein toxicity stress, ultimately leading to cell death. In this paper, we provide a concise overview of the current understanding concerning the metabolism of copper, copper-related diseases, the hallmarks of copper toxicity, and the mechanisms that regulate copper toxicity. Additionally, we discuss the implications of cuproptosis mutations in the development of ARD, as well as the potential for targeting cuproptosis as a treatment for ARD.

## 1 Introduction

With the continuous advancement of science and technology, there has been a significant increase in life expectancy, leading to a rise in the prevalence of ARD worldwide. This global aging trend has resulted in an escalating social and economic burden, placing aging research at the forefront of attention (Chakravarti et al., 2021). The growing elderly population has exposed the challenges faced by human society in terms of life and health, as ARD gradually emerge as a perilous cycle that threatens the lives of older individually (Belikov, 2019). Age itself remains the primary cause of ARD, as nearly all such diseases and mortality rates experience an exponential increase with advancing age. This phenomenon, often referred to as intrinsic aging in certain studies, accelerates the decline of cellular and tissue homeostasis associated with aging (Krisko and Radman, 2019). The specific pathogenesis underlying ARD is still unclear, hampering the diagnosis and treatment of these conditions. Nevertheless, these diseases exhibit shared fundamental mechanisms, including oxidative stress, inflammation, accumulation of metal ions, protein damage, cellular impairments, and functional disorders (Luo et al., 2020; Mazhar et al., 2021).

Oxidative stress, characterized by an imbalance between the production of reactive oxygen species (ROS) and the body’s antioxidant defense mechanisms, serves as a central hallmark of ARD. Copper, an essential cofactor for antioxidant enzymes such as superoxide dismutase, plays a dual role. While vital for neutralizing ROS, excessive copper levels can disrupt this balance, leading to increased oxidative stress and cellular damage, which is a common thread in various ARD, including prominent neurodegenerative conditions such as Alzheimer’s and Parkinson’s diseases (PD; Huang, 2023). Chronic, low-grade inflammation, termed “inflammation, “is a defining feature of aging and contributes to age-related ailments. Copper, through its facilitation of immune cell activation and subsequent release of pro-inflammatory cytokines, is emphasized for its propensity to simulate pro-oxidative and pro-inflammatory attributes. These characteristics are prominently associated with the emergence of cardiovascular diseases, neurodegenerative disorders, and various other conditions related to aging (Pereira et al., 2016).

Metal ions, serving as vital trace nutrients for human beings, play a significant role in human health by participating in the composition of crucial proteins and enzymes. However, an excess or insufficiency of essential metal ions can result in cellular damage (Tang et al., 2022). For instance, iron death, a form of iron-dependent regulated cell death driven by uncontrolled lipid peroxidation, can occur due to imbalanced iron levels (Gan, 2021). Similarly, copper, an indispensable cofactor for all living organisms, also has its drawbacks. Transition metals like copper and iron possess multiple oxidation states, with their reduced forms represented as Cu^+^ and Fe (II), respectively. This redox capability renders them valuable in various energy production processes but also allows them to catalyze the generation of harmful ROS (Brewer, 2010). Furthermore, if their concentration surpasses the threshold maintained by the evolutionarily conserved steady-state mechanism, they directly bind to the acylation component of the TCA cycle, leading to protein aggregation and subsequent loss of iron–sulfur cluster proteins (Tsvetkov et al., 2022). This ultimately results in protein’s hydrotoxic stress and triggers cell death. Nonetheless, cellular copper concentration is actively maintained at a low level through a steady-state mechanism that operates across concentration gradients to prevent the accumulation of harmful free copper inside cells. Surprisingly, recent research by Tsvetkov and colleagues demonstrated that intracellular copper induces a distinct form of cell death compared to that caused by oxidative stress, suggesting an expanded steady-state mechanism for copper (Tang et al., 2022).

Further investigations are warranted to elucidate specific mechanisms and develop effective diagnostic and therapeutic strategies for ARD. During the aging process, the human body undergoes changes in copper content, particularly in pathological tissues where the normal copper homeostatic system is disrupted. This disruption results in the production of destructive ROS and a gradual loss of organ function. Notably, a significant decrease in copper levels is observed in PD nigral degeneration, which is characterized by dopaminergic neurodegeneration in the main site leading to clinical motor disorders (Surmeier et al., 2017). The disruption of copper homeostasis and the subsequent accumulation of copper in specific tissues can initiate cellular dysfunction, particularly notable in conditions like Wilson’s disease, where impaired copper transport results in excessive copper in the liver and brain, leading to severe neurological consequences (Bandmann et al., 2015). The accumulation of metal ions not only disrupts cellular balance but also acts as a precipitating factor in disease progression. Copper’s redox properties can lead to protein aggregation and oxidative modification, a characteristic feature in several age-related neurodegenerative diseases such as Alzheimer’s and Parkinson’s (Chen et al., 2023). These abnormal protein aggregates and misfolded species, attributed to copper-induced oxidative stress, often exert neurotoxic effects and hinder cellular function. The maintenance of copper homeostasis is vital for optimal cellular function, and disruptions in copper metabolism can compromise mitochondrial integrity (Ruiz et al., 2021), affecting energy production and causing cellular dysfunction. Such disturbances are implicated in ARD where cellular vitality and energy generation are compromised, including cardiovascular disorders and muscular degeneration. ARD frequently lead to functional impairments in various organ systems, with copper playing a crucial role as a cellular regulator. Dysregulated copper homeostasis can result in functional abnormalities within the brain, heart, ocular structures, and other vital tissues, contributing significantly to the clinical manifestations of these age-related ailments (Chen et al., 2022). Age-dependent changes in copper levels also seem to contribute to the onset of other neurodegenerative diseases, such as Alzheimer’s disease (AD), Huntington’s disease (Scheiber et al., 2014). Furthermore, disrupted copper homeostasis is observed to varying degrees in ARD, including coronary heart disease, heart failure, cardiomyopathy, diabetic retinopathy, age-related macular degeneration, and muscular atrophy (Ford, 2000; Aydin et al., 2005; Klevay, 2006; Melnikov et al., 2014; Nabi et al., 2021; Lin et al., 2022).

The plasma copper level holds promise as a potential biomarker for copper-related mortality and a crucial pathogenic factor for ARD. Therapeutic strategies aimed at modulating copper homeostasis have shown substantial efficacy in mitigating the incidence and advancement of these conditions, underscoring the potential of copper homeostasis as a viable therapeutic avenue. While previous studies have predominantly focused on cancer, this article provides a comprehensive summary of the current understanding of the mechanism of copper ptosis, its functional role in the development of ARD, and its potential implications in pharmacology and therapeutic interventions for ARD.

## 2 Characteristics of copper metabolism

### 2.1 Copper homeostasis regulation in cells

Copper is of paramount importance in numerous fundamental processes necessary for the maintenance of optimal human health. These critical functions encompass cellular respiration, the formation of connective tissues, effective wound healing, energy metabolism from macronutrients, synthesis of catecholamines, and the modulation of iron flow. Consequently, the meticulous control of copper concentrations within cells and tissues remains indispensable (Menkes et al., 1962; Compston, 2009). Sustaining the intricate equilibrium of copper within the body hinges on a pair of adenosine triphosphatases (ATPases) situated in cell membranes, denoted as ATP7A and ATP7B. Inactivating mutations in ATP7A and ATP7B lead to Menkes disease and Wilson disease (WD), respectively. Menkes disease is a fatal genetic disorder characterized by systemic copper deficiency due to impaired absorption of dietary copper (Menkes et al., 1962; Kodama et al., 1999; Monty et al., 2005; Ravia et al., 2005). On the other hand, WD results from hepatic inability to eliminate excess copper from circulation, leading to systemic copper overload (Lutsenko et al., 2007; Compston, 2009). ATP7A and ATP7B play indispensable and complementary roles in the movement and dispersion of copper within cells and between various tissues (Lutsenko et al., 2007). Both transporters play critical roles in the digestive tract. ATP7A facilitates the release of copper from absorptive cells (enterocytes) to transport dietary copper into the bloodstream (Monty et al., 2005; Ravia et al., 2005). Meanwhile, ATP7B, operating within the enteric system, stores copper within intracellular vesicles, thereby upholding copper equilibrium, a vital component of normal lipid regulation in the absorptive epithelium (Pierson et al., 2018).

Dietary copper initially exists in the Cu^2+^ oxidation state. At the cell’s brush border, enzymes reduce Cu^2+^ to Cu^+^ before it enters the cell (Ohgami et al., 2006). Reduced copper is then bound by copper chaperones, with Atox1 being one such chaperone that delivers copper to ATP7A and ATP7B (Hamza et al., 2003). Under conditions of low copper levels, ATP7A resides in the trans-Golgi network (Nyasae et al., 2007). However, when intracellular copper levels rise, ATP7A relocates to the basolateral membrane, facilitating the transport of Cu^+^ into the portal circulation. Simultaneously, ATP7B sequesters copper within vesicles, presumably to manage cytosolic copper levels or transport copper to copper-dependent enzymes situated within these vesicles.

### 2.2 Normal process of copper metabolism in human body

#### 2.2.1 Digestive system

Dietary copper is absorbed by the intestinal mucosa and transported to the liver and kidneys through the portal blood. The liver releases most of the absorbed copper into the bloodstream (Turnlund, 1998), It has been demonstrated that the uptake of dietary copper is regulated (Lönnerdal, 2008). Fluctuating levels of dietary copper not only influence the general flow of copper within the intestine but also impact the accumulation of copper within the epithelial cells of the intestine (Arredondo et al., 2000). Consequently, copper is conveyed from the liver (and kidney) to other tissues through ceruloplasmin. After entering the liver and kidney, the freshly absorbed copper becomes integrated into diverse compartments. These include intrinsic copper enzymes, secreted proteins dependent on copper, and in the case of the liver, bile as well (Turnlund et al., 1989).

#### 2.2.2 Central nervous system

Copper plays an indispensable role in the physiology of the central nervous system (CNS). Serving as a cofactor for enzymes such as dopamine-beta-hydroxylase, peptidyl-alpha-hydroxylase, superoxide dismutase, and many others, copper is a key contributor to essential processes within the CNS, including catecholamine biosynthesis, neuropeptide and hormone activation, prevention of ROS, respiration, and other crucial functions for normal CNS operation (Lutsenko et al., 2010). In the brain, copper gains access to neurons through specific Cu-ATPase transport proteins, such as ATP7A and ATP7B, a process that has been extensively validated through animal studies and *in vitro* experiments (Schmidt et al., 2018). Copper is a constituent of superoxide dismutase (SOD), which is critical for protecting neurons from oxidative stress damage. The presence of copper is essential for maintaining SOD’s activity. Accumulation of copper in the brain has been associated with Wilson’s disease, a condition in which copper buildup can lead to neurodegenerative disorders, thus establishing a clear logical connection between copper metabolism and accumulation in the brain (Barnes et al., 2005).

#### 2.2.3 Musculoskeletal system

The main mechanisms by which copper accumulation leads to muscle damage and functional impairment include oxidative stress, inflammatory responses, disruptions in cellular energy metabolism, and apoptosis of muscle cells. Excess copper can induce oxidative stress, which damages the structural integrity of muscle cells. The results of studies involving Copper Nanoclusters (CuNCs) exposure on primary muscle cells and mouse gastrocnemius muscle suggest that exposure to CuNCs may be a risk factor for the skeletal muscle system (Liu et al., 2016). Subsequently, the inflammatory response in cells exacerbates cellular damage. Additionally, copper accumulation can disrupt intracellular copper homeostasis, affecting the regulation and distribution of copper within cells. Furthermore, copper is involved in cellular energy metabolism, and its accumulation may lead to insufficient energy supply, affecting muscle contraction. Copper accumulation of copper in non-CNS tissues occurs in the pre-symptomatic stage of SOD1G93A, representing a potential pathological feature that cannot be solely explained by the overexpression of mSOD1. Since the central nervous system lacks copper uptake, the overexpressed mutant SOD1 in these tissues may be deficient in copper. Elevated copper concentrations in muscle tissues have also been found to be pathological and not merely a result of SOD1 overexpression (Enge et al., 2018). Ultimately, copper accumulation may induce apoptosis in muscle cells, leading to muscle loss and functional impairment. The specific impact of these mechanisms can vary depending on the degree of copper accumulation and individual differences.

#### 2.2.4 Cardiovascular system

The disruption of copper homeostasis is associated with the pathogenesis of chronic cardiovascular disease (CVD). CVDs are typically mediated by alterations in lipid metabolism resulting from the generation of ROS (Palinski et al., 1990). Research has shown a strong correlation between elevated copper levels, catalyzing the production of free radicals, and low-density lipoprotein (LDL), which is a significant contributor to cardiovascular diseases (Ford, 2000). Extensive evidence from prospective studies suggests a close association between elevated serum copper levels and conditions such as myocardial infarction, atherosclerosis, diabetes-related complications, and mortality resulting from ischemic heart disease (Kok et al., 1988; Salonen et al., 1991), highlighting the potential role of copper in the pathophysiology of cardiovascular diseases. The heart is one of the organs most sensitive to the generation of ROS, which can impact biomolecules, leading to changes in oxidative/antioxidative status and ultimately causing irreversible damage to cardiac tissues and myocardial cells (Yang et al., 2020). Although copper-induced cardiac damage can result in oxidative injury and tissue damage, it has not been widely reported. Structural and morphological changes in cardiac tissues are triggered by oxidative stress and inflammatory responses following copper exposure.

**Figure 1:**
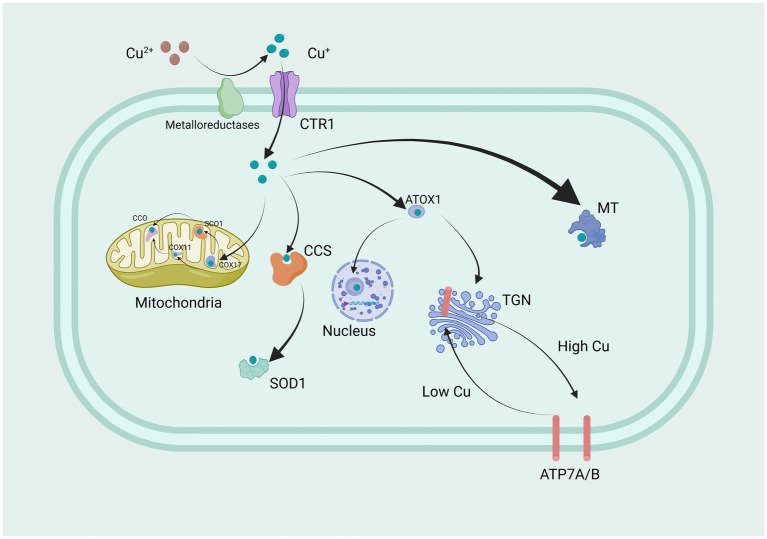
Copper transport and regulation mechanisms in cells.

It is widely accepted that cells in most organs acquire copper from circulation primarily through the high-affinity copper transporter CTR1, located at the basolateral membrane (Lee et al., 2002). Copper is delivered to the extracellular N-terminal domain of hCTR1, where it is guided into a pore. The N terminus and pore contain essential methionine residues involved in this process. Copper binding at the outer surface of hCTR1 appears to induce a conformational change in the cytoplasmic loop, although the purpose of this change is not yet understood. Differences in protein size and abundance recognized by anti-hCTR1 antibodies on SDS-PAGE gels have contributed to uncertainties regarding hCTR1 expression patterns and levels in different cells and tissues. The copper ion passes through the pore and exits at the inner surface, possibly bound at the carboxyl terminus for delivery to its target proteins, which serve as key regulators (Maryon et al., 2007). The understanding of the mechanism and role of hCTR1 has rapidly advanced in recent years (Figure 1).

Extracellular Cu^2+^ is reduced by metal ion reductases to Cu^+^, which is then transported into the cell through CTR1, a highly specific protein for copper uptake. Within the mitochondria, Copper is conveyed by COX17 to copper-binding proteins SCO1, SCO2, and COX11, thereby assisting in their conveyance to CCO, thereby triggering enzyme activity within the respiratory chain. Simultaneously, CCS conveys copper to SOD1, engaging in antioxidant functions. Furthermore, ATOX1 also transports a portion of copper to the nucleus of the cell. Here, it binds to transcription factors, ultimately regulating gene expression. Another portion of copper is localized in the trans-Golgi network (TGN) by the copper transport ATPase, which pumps copper from the cytoplasm into the TGN. Moreover, when intracellular copper levels increase, these copper transport proteins are also involved in transporting copper to the cell membrane, controlling the copper homeostatic environment inside and outside the cell. This is a crucial point, as mutations causing their inactivation lead to Menkes disease and Wilson disease (WD). Copper is delivered to specific proteins by copper chaperones or chelated by metallothioneins (MT) for storage. CTR1, Copper Transporter 1; COX17, cytochrome c oxidase copper chaperone 17; SCO1, synthesis of cytochrome c oxidase 1; COX11, cytochrome c oxidase copper chaperone 11; CCO, cytochrome c oxidase; CCS, copper chaperone for superoxide dismutase; SOD1, superoxide dismutase 1; ATOX1 antioxidant 1 copper chaperone; TGN, trans-Golgi network; MT, Metallothioneins; ATP7A and ATP7B, ATPase copper transporter 7A and 7B.

## 3 Occurrence and mechanism of intracellular cuproptosis

A recent study by Tsvetkov et al. introduced a novel form of cell death induced by copper. The scientists observed that an excess of intracellular copper results in the accumulation of lipoylated dihydrolipoamide S-acetyltransferase (DLAT), an enzyme linked to the mitochondrial TCA cycle. This aggregation results in proteotoxic stress and triggers a unique type of cell death (Tsvetkov et al., 2022). Another report described a distinct form of regulated cell death induced by copper, referred to as “cuproptosis” The authors provided evidence that an overabundance of intracellular copper, achieved through the use of copper ionophores or copper importers, activates cell death pathways involving mitochondrial iron–sulfur protein (Fe-S) ferredoxin 1 (FDX1). FDX1 functions as a reductase, transforming Cu^2+^ into the more hazardous Cu^+^ form (Duan and He, 2022). The study further identified that cuproptosis is characterized by the aggregation of mitochondrial lipoylated proteins, leading to cell death that is different from apoptosis or necrosis. Interestingly, the researchers found that elesclomol treatment, which typically activates caspase-3, a key player in apoptosis, did not induce caspase-3 activation in copper-induced cell death. Furthermore, blocking the apoptotic pathway or other recognized programmed cell death pathways failed to avert copper-induced cell demise, suggesting a distinct mechanism (Wang et al., 2022). The precise morphological attributes associated with cell death induced by copper remain uncertain, as it does not exhibit the typical features associated with programmed cell death, such as nuclear fragmentation in apoptosis or mitochondrial contraction in ferroptosis. This suggests the possibility of a novel cell death mechanism different from previously known patterns. Given the involvement of copper and copper-induced cell death in various diseases, understanding the initiation and regulatory mechanisms of this process holds significant therapeutic implications.

### 3.1 Ferredoxin 1

To further elucidate the relationship between cell death induced by copper and the TCA cycle, the researchers examined the screening outcomes of a metabolic gene sgRNA library, affirming a robust connection between FDX1 (ferredoxin 1), a direct target of the copper ion carrier, and LIAS and DLAT, two established proteins susceptible to lipoylation. Interestingly, it was observed that cells with high dependence on mitochondrial respiration exhibited greater sensitivity to copper-induced cell death (Wang et al., 2022). Copper ion carriers, such as elesclomol, are small molecule transport proteins that facilitate the entry of copper ions into cells. These carriers serve as valuable tools for exploring the mechanisms underlying copper ion cytotoxicity (Morgareidge and Hammel, 1975). Cells that depend on active mitochondrial respiration exhibit a greater vulnerability to elesclomol treatment in contrast to those reliant on anaerobic glycolysis. The buildup of copper in vital elements of the TCA cycle triggers cell demise, and FDX1 serves as an upstream controller of protein lipoylation. FDX1 knockout experiments demonstrated that its absence reduced the lipoylation of DLAT and DLST (components of the TCA cycle) and prevented protein acylation, resulting in decreased sensitivity of cells to copper-induced cell death. Furthermore, it was demonstrated that DLAT and DLST lose their ability to bind copper following the depletion of FDX1 (Li et al., 2022). Acylation is essential for copper binding, which indirectly clarifies the copper-DLAT binding and subsequent acylation-dependent DLAT oligomerization. Abnormal oligomerization of DLAT induces the formation of DLAT lesions, leading to increased levels of insoluble DLAT, protein toxicity, and cell death. As mentioned earlier, FDX1 is involved in regulating protein acylation and also reduces Cu^2+^ to Cu^+^, inhibiting the synthesis of Fe-S clusters and disrupting the production of Fe-S cluster proteins. Similarly, the use of the copper ion carrier elesclomol, under FDX1 regulation, inhibits the synthesis of Fe-S clusters and reduces the steady-state levels of Fe-S cluster proteins (Tang et al., 2022). However, it remains unclear whether this process promotes copper-induced cell death. Existing research is progressively approaching the core of the mechanism behind copper-induced cell death. It is also possible that other metal ions induce distinct forms of cell death beyond the details associated with copper-induced cell death.

Concerning the exact mechanism through which copper ions trigger cell death, multiple hypotheses have been put forth. These include the initiation of apoptosis, caspase-independent cell demise, the production of ROS, and the hindrance of the ubiquitin-proteasome system. Nonetheless, a universally accepted theory remains elusive. Therefore, additional research is imperative to unravel the precise mechanisms governing cell death induced by copper (Tsvetkov et al., 2022).

### 3.2 Glutathione

Glutathione (GSH) serves as an alternative ligand for mediating the delivery of copper to various targets in the body’s tissues. However, excessive copper content in the body can surpass the binding capacity of inert complexes, leading to copper toxicity, oxidative stress, and depletion of reduced GSH. This depletion occurs due to improper binding of cysteine-rich sites, resulting in cellular damage. In Long-Evans Cinnamon rats, an animal model of Wilson’s disease characterized by copper overload, there is a significant accumulation of copper in mitochondria. This accumulation disrupts mitochondrial membrane integrity, depletes GSH stores, and exacerbates oxidative stress-related damage within the organelles (Zischka et al., 2011; Zischka and Lichtmannegger, 2014). To explore the toxicity associated with copper ion carriers, scientists conducted experiments using both pharmacological and genetic strategies aimed at blocking various cell death pathways. These approaches targeted apoptosis (utilizing the caspase inhibitor Z-VAD-FMK or dual knockdown of BAK and BAX), ferroptosis (employing ferrostatin-1 and lipstatin-1), and necroptosis (via necrostatin-1). Intriguingly, these interventions proved ineffective in preventing cell death induced by ES–Cu complexes across multiple cancer cell lines. Notably, the hydrophilic antioxidant GSH exhibited the ability to alleviate the toxicity of ES–Cu complexes by binding to intracellular copper. However, other antioxidants, including N-acetylcysteine, α-tocopherol, ebselene, and JP4-039, were incapable of rescuing the growth inhibition induced by ES–Cu. These findings suggest that ROS, encompassing mitochondrial ROS, do not play a pivotal role in cuproptosis (Tang et al., 2022).

**Figure 2:**
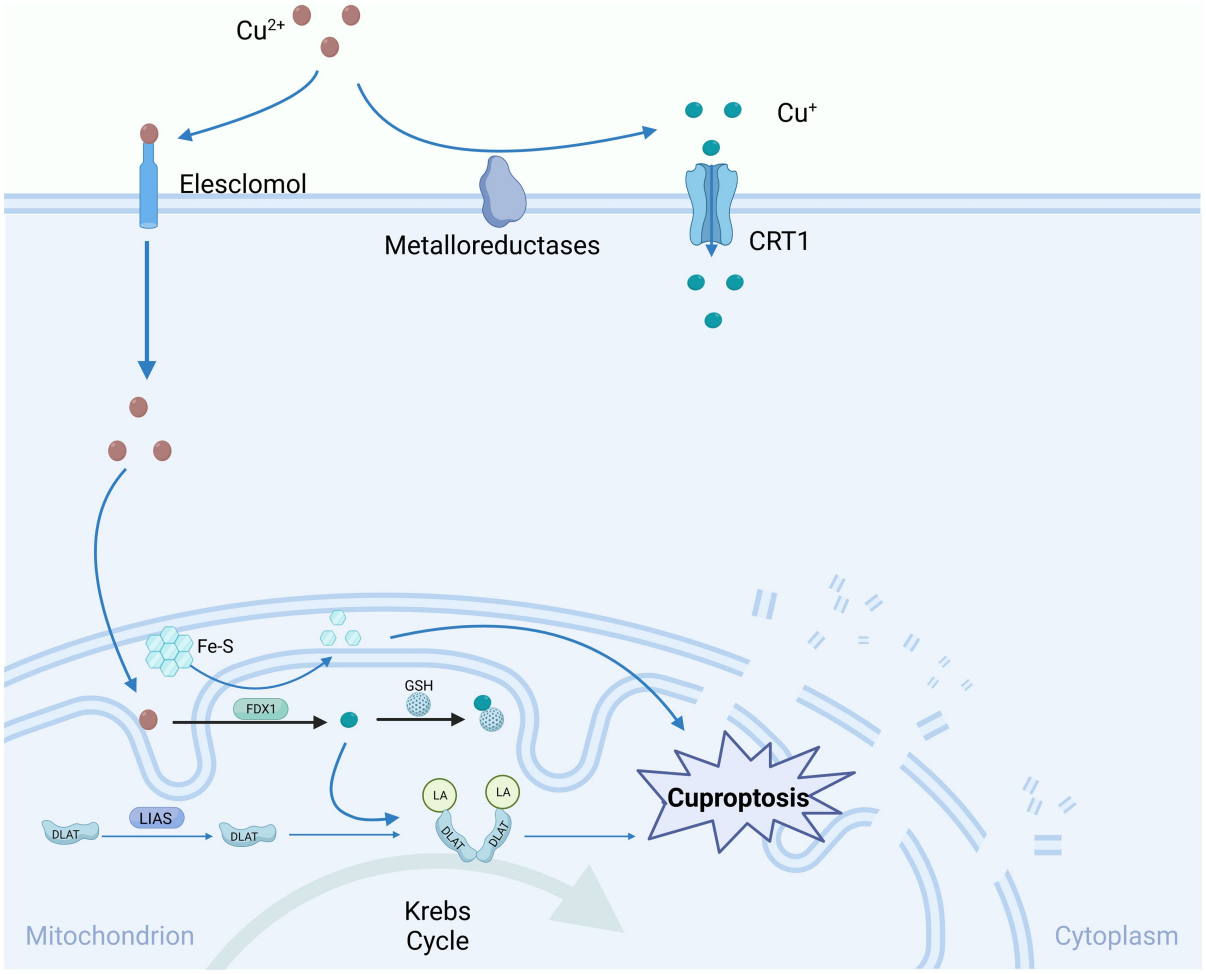
Copper-induced cell death mechanisms and mitochondrial dysfunction.

Certain researchers have proposed that mitochondrial GSH, serving as an innate intracellular copper escort, retards copper-induced cell death by impeding lipoylation and encouraging the oligomerization of DLAT (Li et al., 2022). Evidence indicates that mitochondrial GSH functions as a reductase, reducing oxidative phosphorylation, and the depletion of mitochondrial GSH precedes the generation of ROS and activation of procaspase 3, which is a critical process in apoptosis (Armstrong et al., 2002). However, further investigation is needed to establish a clear connection between the inhibition of cuproptosis by GSH and the underlying mechanisms of cell death (Figure 2).

Cu-induced cell death predominantly targets mitochondria, resulting in oxidative damage to mitochondrial membranes and the compromised function of enzymes within the TCA cycle. Elesclomol attaches to extracellular Cu^2+^ and conveys it into the cell, where it undergoes reduction to Cu^+^ via the expression of FDX1. Consequently, these lipidated proteins trigger the aggregation of dihydrolipoamide S-acetyltransferase (DLAT). FDX1/LIAS, acting as an upstream regulator of protein lipidation, promotes the loss of DLAT and Fe-S clusters, collectively contributing to copper-induced cell death. One such lipidated protein is directly associated with DLAT in the pyruvate dehydrogenase complex, where copper can facilitate the lipidation of DLAT through disulfide bond-dependent aggregation. Gene knockout of FDX1 or LIAS reduces protein lipidation and inhibits copper-induced cell death. Glutathione, a sulfur-containing thiol, acts as a copper chelator, blocking copper deposition. Copper chelators like TTM can also inhibit copper sedimentation. CTR1, Copper Transporter 1; Fe-S, iron–sulfur clusters; FDX1, Ferredoxin 1; GSH, Glutathione; DLAT, Dihydrolipoamide Acetyltransferase; DLAT-LA, Dihydrolipoamide acetyltransferase lipoylation; LIAS, Lipoate Synthase.

### 3.3 High mobility group box 1

High Mobility Group Box 1 (HMGB1) is a nuclear protein that is not associated with histones that functions as a damage-associated molecular pattern during cell death (Chen et al., 2023). It is the most extensively studied member of the high-mobility group (HMG) protein family and functions as a detector and coordinator of cellular stress responses, as well as a DNA partner, chromatin guardian, autophagy maintainer, and guardian of apoptotic cell death in the nucleus and cytoplasm of cells. It plays a crucial role, especially in age-related chronic diseases (Taverna et al., 2022). HMGB1 is particularly important in age-related chronic diseases. In a mechanistic study, it was discovered that cells undergoing cuproptosis release high-mobility group box 1 (HMGB1), which acts as a damage-associated molecular pattern and initiates inflammation (Liu et al., 2022). In the process of inducing mitophagy, the clustering of impaired mitochondria results in decreased ATP production and an elevated generation of ROS (Bock and Tait, 2020). Although mitochondrial ROS can trigger cell apoptosis as signal molecules, this pathway is not significant in the pathogenesis of hemochromatosis (Tsvetkov et al., 2022).

The accumulation of copper induces depletion of adenosine triphosphate (ATP), which mechanistically activates AMP-activated protein kinase (AMPK), resulting in phosphorylation of HMGB1 and increased extracellular release. Conversely, inhibition of AMPK activation, either through genetic inhibition (using RNAi) or pharmacological inhibition (using dorsomorphin), limits cuproptosis and the release of HMGB1. Functionally, cuproptotic cells deficient in HMGB1 show a reduced ability to promote the production of inflammatory cytokines through the advanced glycosylation end product-specific receptor (AGER, also known as RAGE; Liu et al., 2022). This research is in accordance with earlier discoveries indicating that copper-induced cell death, characterized as a type of metabolic demise, encompasses not only the degradation process of impaired proteins but also perturbed energy homeostasis. Nonetheless, the AMPK-mediated autophagy pathway could potentially influence the accumulation or breakdown of detrimental mitochondrial proteins, creating a feedback mechanism that governs susceptibility to hemochromatosis (Herzig and Shaw, 2018).

### 3.4 P53 gene

Cuproptosis represents an innovative variant of copper-induced cell death, primarily observed in cells that predominantly rely on oxidative phosphorylation as their primary metabolic route for generating energy. Intriguingly, the tumor suppressor protein p53 emerges as a vital metabolic controller. It suppresses glycolysis while promoting the metabolic transition toward oxidative phosphorylation, thereby increasing the susceptibility of cells to copper-induced cell death (Kruiswijk et al., 2015). Furthermore, p53 disrupts several pivotal catalytic reactions within glycolysis. As an example, p53 hinders the transformation of glucose into glucose-2-phosphate by facilitating the degradation of HK6 mRNA (Wang et al., 2014) and p53 inhibits PFK1 activity by promoting PFKFB1/6 and TIGAR expression, leading to fructose-6-phosphate inhibition. Additionally, p53 decreases PGAM29 protein levels and suppresses ENO30 expression, inhibiting the conversion of 3-phosphoglycerate to 2-phosphoglycerate and 1-phosphoglycerate to phosphoenolpyruvate, respectively. These regulatory mechanisms effectively fine-tune glycolysis to meet cellular metabolic demands (Ruiz et al., 2021). In the ultimate phase of glycolysis, p53 suppresses the expression of lactate dehydrogenase A (LDHA) through either direct binding to the LDHA promoter or by stimulating the degradation of the LDHA transcription factor HIF-1α (Pan et al., 2022). This inhibition causes an accumulation of pyruvate in the cell, which disrupts its involvement in the TCA cycle and oxidative phosphorylation. These pathways are critical for copper-induced cell death, meaning that p53’s inhibition of LDHA expression may contribute to cell death in response to copper exposure. Under glucose limitation, the cellular ATP level decreases, leading to an increase in the AMP/ATP ratio, which further activates the AMPK signaling pathway (Semenza, 2010). The activation of AMPK triggers the phosphorylation and activation of p53 (Herzig and Shaw, 2018). This establishes a feedback loop between p53 and glucose metabolism, further reinforcing the shift from glycolysis to oxidative phosphorylation. The sustained activation of AMPK triggers autophagic cell death by inhibiting mTOR (Jones et al., 2005; Delavallée et al., 2011). Therefore, copper-induced cell death may occur simultaneously with autophagic cell death in response to glucose restriction in the fly disease.

p53 plays a significant role in sensitizing cells to copper-induced cell death by modulating glycolysis and promoting mitochondrial metabolism. Importantly, p53 also exerts an impact on the production and functionality of iron–sulfur clusters and copper chelators such as GSH, which are pivotal elements in the pathway of copper-induced cell death. Previous research has demonstrated a connection between copper homeostasis and p53-mediated cell apoptosis (Formigari et al., 2013). Physiological copper levels can directly interact with p53, inhibiting its ability to bind to DNA (Hainaut et al., 1995). Conversely, elevated copper levels activate p53, resulting in apoptosis of cancer cells (Narayanan et al., 2001). Copper accumulation can contribute to the development of non-cancerous conditions such as Wilson’s disease, neurodegenerative diseases, and cardiovascular diseases (Ahuja et al., 2015; Chen et al., 2020).

p53 is implicated in the promotion of cuproptosis through the enhancement of mitochondrial metabolism. Studies have shown that p53 activity is necessary for maintaining mitochondrial integrity and function, as p53 mutations or deficiency result in reduced mitochondrial content, cytochrome c oxidase (COX) activity, and respiratory metabolism (Zhou et al., 2003; Saleem et al., 2009). This suggests that p53 may play a role in autophagy. Additionally, p53 promotes the activation of the pyruvate dehydrogenase (PDH) complex by facilitating phosphorylation, a crucial component of the TCA cycle essential for cuproptosis (Zhang et al., 2011; Contractor and Harris, 2012; Tsvetkov et al., 2022). Moreover, p53 transcriptionally activates GLS2, which promotes the conversion of glutamine to glutamate (Hu et al., 2010; Suzuki et al., 2010). Remarkably, in conditions of glutamine scarcity, p53 promotes the upregulation of the aspartate/glutamate transporter SLC1A3. This serves to sustain aspartate metabolism and facilitate the operation of the TCA cycle (Tajan et al., 2018). These discoveries imply that p53 might enhance the sensitivity of cells to cuproptosis by stimulating the TCA cycle. Furthermore, p53 may enhance oxidative phosphorylation to increase cell death through cuproptosis. As an illustration, p53 triggers the upregulation of SCO2 expression, facilitating mitochondrial respiration essential for COX complex assembly. Conversely, depletion of SCO2 instigates a metabolic shift toward glycolysis, mirroring observations in p53-deficient cells (Delavallée et al., 2011).

Iron–sulfur (Fe-S) clusters are essential protein cofactors involved in various biological processes, including enzyme catalysis, electron transfer, and metabolic stress sensing. Considering p53’s role in governing the expression of multiple genes associated with the biogenesis of Fe-S clusters, it is plausible to suggest that this mechanism may represent another pathway by which p53 regulates cuproptosis. This hypothesis warrants additional scrutiny. One example of such a gene is FDXR, which is responsible for encoding the iron–sulfur protein reductase. This enzyme plays a crucial role in facilitating the transfer of electrons from NADPH to FDX1/2 and, subsequently, to cytochrome P450, thereby contributing to the process of Fe-S cluster biogenesis (Sheftel et al., 2010). The inhibition of HSPA9 leads to a reduction in both mitochondrial membrane potential and content. This phenomenon has the potential to disrupt the process of Fe-S cluster biogenesis, as HSPA9 serves as a critical partner protein in the assembly of Fe-S clusters (Schilke et al., 2006). These findings suggest that p53 may coordinate cuproptosis through the regulation of Fe-S cluster biogenesis and homeostasis, although its involvement in Fe-S cluster instability remains to be elucidated.

In addition, p53 regulates the levels of the endogenous copper chelator GSH. GSH not only serves as a crucial defense against oxidative stress but also acts as a copper chelator. Depletion of GSH, caused by buthionine sulfoximine, has been shown to promote copper-induced cell death consistently, as demonstrated by Tsvetkov et al. (Chung et al., 2019). P53 transcriptionally suppresses the expression of SLC7A11, which is essential for cysteine uptake, a precursor for GSH, resulting in decreased GSH levels, increased lipid ROS, and ferroptosis (Jiang et al., 2013). These discoveries indicate a potential shared surveillance mechanism for cell death induced by copper and iron, which involves the pivotal role of GSH. Notably, p53 serves to counteract this mechanism and, intriguingly, promotes GSH biosynthesis by activating various metabolic genes, including TIGAR (Hu et al., 2010), GLS4, and SESN6/3, to protect cells from oxidative toxicity. P53’s role in copper-deficient conditions may vary due to its differential regulation of GSH biogenesis. A plausible hypothesis is that p53 functions as an intracellular gauge for monitoring copper concentrations, ensuring the maintenance of appropriate physiological copper levels. It accomplishes this by averting the buildup of excessive, labile copper and eliminating cells afflicted by copper-induced damage, especially when copper uptake or levels are altered due to pathological or experimental conditions. This notion gains partial support from previous investigations that have documented p53’s dual roles, displaying both pro-survival and pro-death activities in response to different intensities of stress signals (Delavallée et al., 2011; Kruiswijk et al., 2015). Overall, p53 may prevent or promote cuproptosis by divergently regulating the biogenesis of the copper chelator GSH under physiological or copper-excessive conditions.

## 4 Cuproptosis and copper homeostasis in ARD

There is mounting evidence indicating a significant contribution of copper ptosis to the pathogenesis of various ARD, including neurodegenerative diseases, cardiovascular diseases (CVDs), ocular diseases, and motor system diseases, as shown in Table 1

**Table 1:** Signs of copper and Cuproptosis in ARD.

Diseases	Change of copper content	Research model	Research mechanism	References
NDs				
AD	Cu↑	Human	NA	Hureau (2023)
	Cu↑	*In vitro* model	Cu binds to Aβ↑	Esmieu et al. (2019)
	Cu↑	Human	ROS↑	Sensi et al. (2018)
	Cu↑	Rabbit model	Copper promotes ROS production and affects Aβ peptide aggregation	Winner et al. (2011)
PD	Cu↓	*In vitro* model	SOD1	Bisaglia & Bubacco (2020)
	Cu↑	Rat model	thiol modifications induced by copper	Boll et al. (2008)
	Cu↑	Human	NA	Binolfi et al. (2006)
	Cu↑	*In vitro* model	Promotes misfolding and aggregation of amyloidin	Olivieri et al. (2011)
	Cu↑	*In vitro* model	Cu binding to α-synuclein promotes aggregation and cytotoxicity	Trist et al. (2017)
	Cu↓	Mouse model	Silencing the copper transporter Ctr1 reduces alpha-synuclein aggregation	Trist et al. (2018)
	Cu↑	*In vitro* model	Neuroinflammation and mitochondrial autophagy dysfunction mediated by reactive oxygen species (ROS) and nuclear factor-κB (NF-κB)	Yamamoto & Gaynor (2004)
ALS	Cu↓	Mouse model	Impaired SOD1 interaction	Williams et al. (2016)
	Cu↑	Mouse model	Cu-containing compounds reduce the level of G37R mutant SOD1	Tokuda et al. (2015)
	Cu↓	Mouse model	Bonded copper	Schaufelberger (2019)
CVDs				
Cardiomyopathy	Cu↑	Mouse model	AGEs increase intracellular copper accumulation	Cooper et al. (2004)
	Cu↓	Rat model	Combined accumulated copper	Voziyan et al. (2003)
HF	Cu↑	*In vitro* model	Acylation of tricarboxylate circulating protein induced by copper overload	Grammer et al. (2014)
	Cu↓	Rat model	Copper chelation restores mitochondrial function	Bomer et al. (2022)
	Cu↓	Mouse model	Beta-adrenergic receptor (beta-AR) signaling	Bagheri Varzaneh et al. (2017)
	Cu↓	Rat model	Restore the expression of HF key mitochondrial biogenic regulators	Voziyan et al. (2003)
	Cu↑	Mouse model	Suppressor gene-induced heart-specific MT-IIa overexpression in mice has been implicated in progression from cardiac hypertrophy to heart failure during copper deficiency	Falk (2006)
Atherosclerosis	Cu↑	Human	Elevated copper levels regulate lipid metabolism, low-density lipoprotein oxidation, and inflammatory responses	Zhao et al. (2021)
	Cu↑	Rabbit model	Targeting specific copper supplements preventing endothelial cell death	Koksal et al. (2007)
	Cu↓	Mouse model	Decreased lipolysis activity and increased triglyceride levels	Nakano et al. (2004)
Stroke	Cu↑	Mouse model	Copper can inhibit EPC function by increasing Thrombospondin-1 levels	Xu et al. (2022)
	Cu↑	Human	NA	Cho et al. (2018)
Other ARD				
DM	Cu↑	Mouse model	AGEs may promote cardiotoxicity caused by copper overload	Eshak et al. (2018)
	Cu↑	*In vitro* model	SLC31A1	Duan & He (2022)
	Cu↑	Human	NA	Yin et al. (2019)
Sarcopenia	Cu↑	*In vitro* model	ROS dependent apoptosis	Nagai et al. (2012)
AMD	Cu↑	Human	NA	Aloysius Dhivya et al. (2020)
	Cu↑	Zebrafish model	NA	Wu et al. (2023)

ARD, ARD; NDs, neurodegenerative diseases; AD, Alzheimer’s disease; PD, Parkinson disease; ALS, amyotrophic lateral sclerosis; CVDs, cardiovascular diseases; HF, heart failure; DM, diabetes mellitus; AMD, Age-related macular degeneration. “↑”, upregulation; “↓”, downregulation; “NA”, not application.

### 4.1 Neurodegenerative diseases

#### 4.1.1 Alzheimer’s disease

Alzheimer’s disease (AD) is an advancing neurodegenerative condition that primarily affects older adults (Jalbert et al., 2008). Recent evidence suggests a connection between changes in copper levels and miscellaneous neurodegenerative diseases, particularly AD, where abnormal copper homeostasis is widely acknowledged. Studies examining plasma copper levels in AD patients compared to healthy controls have confirmed elevated copper levels in the AD group, supporting the correlation (Li et al., 2023). Nonetheless, in AD, the significance lies not only in the copper level but also in its distribution between the extracellular and intracellular compartments. The accumulation of copper in the extracellular milieu, especially within amyloid deposits where copper binds to Aβ peptides, holds particular importance (Hureau, 2023). Copper has been shown to influence the aggregation pathway of Aβ, modulating its assembly, making it more toxic, and participating in the generation of ROS involved in oxidative stress, a key event in AD pathogenesis (Esmieu et al., 2019). These findings suggest that copper dyshomeostasis induced by copper accumulation, especially in neurons, contributes to the worsening of AD conditions. Additionally, in the presence of biological reductants such as ascorbate or GSH, copper can undergo redox cycling, leading to the production of ROS and oxidative damage to neurons (Chassaing et al., 2012).

Research indicates that mitochondrial dysfunction, abnormalities in energy metabolism, and oxidative stress are crucial factors in the progression of AD. Consequently, two primary therapeutic approaches are being investigated within the context of AD: symptom-targeting and disease-modifying approaches. Disease-modifying approaches focus on pathways involved in neurodegeneration, such as inhibiting β-and γ-secretases to reduce Aβ production, enhancing Aβ elimination through immunotherapy, and manipulating metal ions to impact aggregation and oxidative stress, High levels of metal ions have been detected in brain areas affected by AD, and the pathological consequences of metal ion-Aβ interactions, such as ROS generation and aggregation, have led to therapeutic strategies aimed at preventing metal ions from binding to Aβ. Copper, in particular, has received significant attention. It plays a role in ROS production due to its redox ability and influences the aggregation of Aβ peptides. Modulating these interactions potentially stabilizes toxic oligomers, which are considered the most harmful entities in the aggregation process (Sensi et al., 2018). Metal-targeted therapies, including copper carriers and chelating agents, have emerged as potential strategies to address copper dyshomeostasis resulting from copper accumulation in AD. Although the exact effects and mechanisms of cuproptosis in the pathogenesis of AD are not fully understood, these findings indicate that targeting cuproptosis holds promise for the development of innovative treatments for AD.

#### 4.1.2 Parkinson’s disease

Parkinson’s disease (PD) stands as the second most common neurodegenerative disorder, following AD in prevalence (Winner et al., 2011). The global prevalence of PD is projected to double by 2040 (Schilke et al., 2006), outpacing the rise in AD incidence (GBD 2015 Neurological Disorders Collaborator Group, 2017). PD is a slowly progressing neurodegenerative condition characterized by motor coordination impairment and non-motor features. Although the precise etiology of PD remains unknown, the disease is primarily associated with environmental exposure to toxins such as iron and copper, which lead to their abnormal accumulation in the brain. The brain exhibits high concentrations of copper ions and other metal ions (Gaier et al., 2013) but in PD, copper levels in brain tissue are reduced, especially in the substantia nigra, the most severely affected area in PD. However, copper levels are found to be elevated in cerebrospinal fluid and blood (Bush, 2000; Bisaglia and Bubacco, 2020). Free copper in cerebrospinal fluid is considered a potential biochemical marker for PD (Boll et al., 2008). *In vitro* studies have demonstrated that micromolar concentrations of various metal ions, including copper, can facilitate the misfolding and aggregation of amyloid-beta protein (Binolfi et al., 2006). Altered copper metallization and aberrant oxidation of copper-binding proteins lead to structural changes, functional impairment, and accelerated degradation, characteristic of degenerated brain regions in PD (Olivieri et al., 2011). Copper binding and oxidation of α-synuclein can disrupt protein stability *in vitro*, triggering misfolding, subsequent degradation, alterations in cellular localization, and accumulation (Carboni and Lingor, 2015). Cell culture models have revealed that copper binding with α-synuclein modulates its cellular localization, promoting aggregation and cellular toxicity (Wang et al., 2010). The activity of protective copper proteins like SOD1 is diminished in the PD brain (Trist et al., 2017), potentially resulting in increased oxidation of α-synuclein and oligomerization in brain regions characterized by heightened oxidative stress. Restoring neuronal copper levels can confer significant neuroprotection in PD and familial amyotrophic lateral sclerosis (FALS), potentially through stabilizing and enhancing the function of disease-relevant copper proteins, including SOD1. Moreover, silencing the expression of Ctr1, a copper transporter, to impede copper uptake reduces α-synuclein aggregation and mitigates neuronal loss in mice (Gou et al., 2021). Conversely, clinical trials have been conducted in PD patients with impaired brain copper levels, leading to functional impairment.

A recent significant finding involves the abnormal accumulation of the major antioxidant enzyme SOD1 in degenerated regions of spontaneously occurring PD brains postmortem. In the presence of copper metabolism disorders, the ability of SOD1 to dimerize and function as an antioxidant is compromised, resulting in a systemic decrease in biologically available copper. This reduction limits the maturation of SOD1 and leads to the accumulation of copper-deficient zinc-containing proteins. Consequently, the intermediate lacking antioxidant activity experiences uncontrolled elevation of free radicals and ROS (Trist et al., 2018). The mechanisms involved in clearing white matter, such as ubiquitin, are closely associated with SOD1 aggregates in SOD1-familial amyotrophic lateral sclerosis (FALS) and PD. These mechanisms are identified based on the shared pathology observed in rare copper metabolism disorders and inflammatory responses linked to neurodegeneration (Greenough et al., 2016). The functional decline of protective copper proteins, such as SOD1, in the PD brain may contribute to increased oxidation of α-synuclein, leading to oligomerization in brain regions characterized by elevated oxidative stress (Trist et al., 2017). Normalizing neuronal copper levels can offer significant neuroprotection in PD and ALS, likely due in part to the stability and function of key disease-related copper proteins, including SOD1. Recent research findings suggest that the accumulation of copper ions in neurons may play a pivotal role in the pathogenesis of PD. Abnormal copper accumulation leads to disruptions in multiple biological pathways, including those associated with ROS and the nuclear factor-κB (NF-κB) pathway. These alterations further culminate in microglia-mediated neuroinflammation and dysfunctional mitochondrial autophagy, exerting a significant impact on the development of PD95 (Zhou et al., 2022). Copper accumulation initially triggers the generation of ROS, a hallmark of oxidative stress, potentially instigating the early activation of the NF-κB pathway. NF-κB typically resides in an inactive form within the cytoplasm, bound to inhibitory κB (IκB) proteins (Yamamoto and Gaynor, 2004). The degradation of IκB proteins leads to the rapid translocation of NF-κB to the nucleus, where it associates with P65 response elements, thereby activating the transcription of inflammatory mediators such as IL-6, TNF-α, and COX2 (Tang and Le, 2016). This pathway plays a pivotal role in the pathogenesis of neuroinflammation associated with PD. Furthermore, sustained copper accumulation adversely affects mitochondria within neurons. Mitochondria are vital centers for cellular energy production, but copper accumulation may result in decreased mitochondrial membrane potential, reduced expression of mitochondrial-related proteins like Parkin and PINK1, and alterations in autophagy-related markers such as P62 and LC3BII/I ratios. Additionally, upregulation of the NLRP3/caspase-1/GSDMD axis may be involved in these processes (Zhou et al., 2022). The interplay of these aberrant pathways culminates in the initiation of an inflammatory response within microglial cells, leading to the release of inflammatory cytokines, including the previously mentioned IL-6 and TNF-α, further exacerbating neuroinflammation. This inflammatory response may exert toxic effects on dopaminergic neurons, ultimately exacerbating the progression of PD. Understanding these copper-related pathways, NF-κB activation, and neuroinflammation is of paramount importance for the development of therapeutic and intervention strategies for PD.

#### 4.1.3 Amyotrophic lateral sclerosis

Amyotrophic lateral sclerosis (ALS) is a neurodegenerative condition characterized by the progressive and rapid degeneration of motor neurons, resulting in muscle weakness, muscle atrophy, and, ultimately, fatality. Approximately 10–20% of ALS cases manifest as familial ALS (FALS), featuring autosomal dominant inheritance patterns. In contrast, the majority of cases, about 80–90%, occur as sporadic ALS (SALS) without any discernible genetic link. One of the main causes of ALS is mutations in the SOD1 gene, which reduce protein folding stability by disrupting metal binding and/or disulfide bond formation, leading to misfolding, aggregation, and ultimately, cellular toxicity (McAlary et al., 2022). Superoxide dismutase 1 (SOD1) is a reliable biomarker of cellular stress (Andersson-Sjöland et al., 2016) that clears superoxide anions and uses copper as a cofactor (Wong et al., 2000). In the realm of ALS, the interplay between CCS and mutant SOD1 leads to a lethal outcome. This interaction diminishes the delivery of copper to mitochondria, facilitating the buildup of unstable SOD1 with an inclination toward acquiring pro-oxidant properties. Consequently, this process triggers toxic gain-of-function effects within motor neurons. Intriguingly, a prior study demonstrated that the overexpression of CCS hastens the deterioration of neurofunction and reduces the lifespan of SOD1G93A mice (Son et al., 2007).

The involvement of copper in the development of ALS is currently under investigation and remains ambiguous. Copper deficiency has been noted to contribute to the anomalous hydrophobic properties of mutant SOD1, resulting in compromised SOD1 interactions and subsequent neuronal toxicity (Gil-Bea et al., 2017). However, copper supplementation has shown potential in ameliorating these effects. In G37R mutant SOD1 mice, a significant portion of mutant SOD1 protein fails to bind to copper. Nevertheless, treatment with Cu-containing compounds reduces the levels of metal-defective SOD1 and increases the pool of SOD1 fully bound to copper (Williams et al., 2016). Furthermore, research has shown that aggregated mutant SOD1 displays reduced copper content in both cultured cells and the spinal cords of transgenic SOD1 mice, irrespective of its capacity to bind copper. Additionally, there is a discovered correlation between the extent of copper deficiency in mutant SOD1 aggregates and the severity of ALS (Gil-Bea et al., 2017). Disruption of cellular copper balance and the compromised activity of Cu-dependent enzymes may also play a role in the detrimental effects of mutant SOD1 protein and the advancement of the disease. In G93A mutant SOD1 mice, an elevation in copper concentration has been noted in skeletal muscle and spinal cord during the pre-symptomatic phase, a condition that worsens as the disease progresses. Furthermore, heightened copper levels have been detected in the cerebrospinal fluid of individuals afflicted with ALS. Treatment with the copper chelator TTM has shown promising results, prolonging survival, reducing motor neuron loss, and alleviating skeletal muscle atrophy in pre-symptomatic and symptomatic SOD1G93A mice. Additionally, d-penicillamine, another copper chelator, has demonstrated beneficial effects in transgenic SOD1 mouse models, delaying disease progression and extending survival (Tokuda et al., 2015). Despite these findings, the role of copper in ALS pathogenesis is still under investigation, and more research is needed to fully understand the complex mechanisms underlying copper dysregulation and its therapeutic implications. By unraveling the precise role of copper and developing targeted interventions, we can potentially advance the development of innovative treatments that may slow down or halt the progression of ALS, bringing hope to patients and their families.

### 4.2 Cardiovascular disease

#### 4.2.1 Cardiomyopathy

Cardiomyopathy is a collection of diseases characterized by structural and functional abnormalities in the myocardium, without evidence of other concurrent diseases that could account for the observed myocardial abnormalities (Schaufelberger, 2019). Its defining feature is inappropriate ventricular hypertrophy or dilation, which, in severe cases, can lead to cardiovascular death or progressive HF (Maron et al., 2006). Myocardial cell loss, resulting from terminal differentiation and cell death, is a major contributor to cardiomyopathy. Various forms of cell death associated with cell loss, including autophagy, apoptosis, and necrosis, have been implicated in myocardial injury (Chiong et al., 2011). Recently, there has been an investigation into the role of copper homeostasis and copper-induced cell death in cardiac pathology, particularly in aging populations. Advanced glycation end products (AGEs) constitute a diverse array of compounds, encompassing over 20 distinct products that result from glycation, a chemical process also referred to as the Maillard reaction. AGEs accumulate in serum and tissues with aging, especially in AGEs induced by sustained hyperglycemia, and play a critical role in cardiac toxicity. Interestingly, AGEs trigger myocardial cell death and exacerbate it when incubated with CuCl_2_ or disulfiram-copper. Moreover, AGEs increase the intracellular accumulation of copper and exhibit characteristics of cuproptosis, such as the loss of Fe-S cluster proteins (FDX1, LIAS, NDUFS8, and ACO2), and reduced lipoylation of DLAT and DLST. These findings suggest that copper overload also contributes to myocardial cell dysfunction. Our study demonstrated that disulfiram has a higher affinity for copper than other metal ions in myocardial cells and exhibits stronger copper toxicity. Copper ion levels in the serum and myocardial tissue were significantly higher in diabetic mice compared to control mice. Dithiothreitol staining revealed increased accumulation of copper salts in myocardial tissue, particularly in the context of diabetes, indicating a potential role for copper in diabetic myocardial injury (Chaudhuri et al., 2018).

In an animal study exploring diabetes-related heart disease, the oral administration of curcumin, a Cu-selective transition metal chelator, for a duration of 7 weeks yielded significant enhancements in myocardial cell structure. This treatment resulted in the partial restoration of cell ultrastructure, improvements in cell volume and alignment, restoration of mitochondrial function, and normalization of the extracellular matrix (ECM; Cooper et al., 2004). Advanced glycosylation end products (AGEs) represent the culmination of non-enzymatic glycation reactions involving proteins. AGEs are considered mediators of ARD and can serve as markers for testing the aging process. Glycation can cause protein cross-linking damage, leading to structural alterations in proteins, resembling those found in aged proteins. AGEs accumulate in the body with age, resulting in increased arterial wall stiffness, disrupted bone metabolism, and the development of osteoporosis. One possible scenario is that AGEs mediate myocardial cell death. Furthermore, Cu-catalyzed oxidative reactions involving Cu^2+^ and Fe^3+^ redox metal ions contribute to the formation of AGEs under *in vivo* conditions. However, these metal ions are sequestered within specific metal transport proteins and other metal-binding proteins in the plasma or cytoplasm (Voziyan et al., 2003). Copper metalloproteins can undergo substantial conformational alterations or even fragmentation subsequent to glycation, resulting in the liberation of bound metals and contributing to a positive feedback loop (Islam et al., 1995; Kang, 2006).

#### 4.2.2 Heart failure

The heart functions as a pump, filling with blood during rest and then contracting to empty, generating a heartbeat. When the heart becomes either too stiff or too weak, it fails to effectively pump enough blood to meet the body’s needs, a condition known as heart failure (HF). HF is a prevalent condition, affecting approximately 40 million individuals worldwide. Several factors can contribute to HF, with the most common risk factors being high blood pressure, coronary artery disease (blockages in the heart’s arteries), diabetes, obesity, smoking, and genetics (Baman and Ahmad, 2020). HF is considered an epidemic disease in the modern world, affecting around 1 to 2% of the adult population. It is a complex, systemic condition triggered by a multitude of factors, including structural, neurohumoral, cellular, and molecular mechanisms that become activated and interconnected after cardiac injury, ultimately leading to excessive volume overload, increased sympathetic activity, circulation redistribution, and the emergence of various clinical signs and symptoms (Tanai and Frantz, 2015).

The mitochondria play a central role in myocardial cell survival and death and are a crucial therapeutic target in HF, closely associated with its pathophysiological processes. HF can be likened to an “engine running out of fuel, “as impaired mitochondrial conversion of energy substrates into ATP (fuel) places a burden on cardiac function. Copper, a trace nutrient, participates in the regulation of mitochondrial bioprocesses, and elevated serum copper levels are significantly associated with HF. Copper overload adversely affects mitochondrial function and worsens HF development. The acylation of TCA cycle proteins induced by copper overload is closely linked to mitochondrial respiration. Copper chelators not only have the potential to treat HF but also partially rescue copper-mediated cell death (Yuan et al., 2022). Studies have found a strong correlation between high copper concentrations and HF and its prognosis (Malek et al., 2006; Alexanian et al., 2014; Grammer et al., 2014). A study conducted on HF rats demonstrated that copper chelation and restoration of muscle cell copper transport resulted in mitochondrial repair and improved cardiac function (Zhang et al., 2020). Nevertheless, the exact mechanism underlying the link between HF and copper overload remains unclear.

In general studies, copper plays a crucial role as a component of cytochrome oxidase (CCO) in mitochondria, and copper deficiency can result in mitochondrial dysfunction (Bomer et al., 2022). Kang et al. conducted a study on HF mice and demonstrated that a 5-week period of dietary copper deficiency led to myocardial dysfunction (Elsherif et al., 2003). They further investigated whether copper supplementation could reverse the HF induced by copper deficiency. The investigation unveiled that mice deficient in copper, when provided with copper supplements for a period of 4 weeks, exhibited a complete restoration of both diastolic and systolic function, along with an improved response to β-adrenergic stimulation (Elsherif et al., 2004). This implies that changes in β-adrenergic receptor (β-AR) signaling could have a pivotal role in the development of HF, and the restoration of β-AR levels might represent a viable therapeutic target (Elsherif et al., 2003). Remarkably, in HF induced by diabetes, disruptions in myocardial copper transport have been noted (Zhang et al., 2020). The control of mitochondrial copper in the hearts of individuals with diabetes exhibited impairment, as evidenced by diminished mRNA and/or protein levels and changes in the mitochondrial distribution of copper chaperones, such as COX17, COX11, and the mitochondrial resident CCS. Intriguingly, the copper chelator TETA was able to restore copper transport in myocardial cells and substantially enhance cardiac function. TETA treatment resulted in the rejuvenation of the compromised myocardial structure and function in diabetic hearts by reinstating copper chaperones and assembly factors for cytochrome c oxidase (CCO). Additionally, the copper ion chelator quinidine also improved cardiac function in animals diagnosed with HF (Bagheri Varzaneh et al., 2017). This suggests that the accumulation of copper ions is inevitable in diabetes-induced HF at some point. Nonetheless, the authors of this study did not elucidate the precise mechanism behind these observations. It could potentially be associated with the inhibition of copper-mediated oxidative stress or the consequences of protein denaturation induced by copper depletion. Consequently, the comprehensive effects of chelators may have relevance to the pathogenesis of HF. Investigating the underlying principles governing this relationship is a promising avenue for future research.

A meta-analysis of 1,504 patients revealed a significant correlation between high serum copper levels and HF (Huang et al., 2019). An increased serum copper/zinc (Cu/Zn) ratio is positively correlated with lung cancer (Wang et al., 2021), aging (Malavolta et al., 2015), pneumonia (Kunutsor et al., 2022) and ischemic heart disease (Laine et al., 2020). Similarly, an elevated serum Cu/Zn ratio was associated with increased HF risk in middle-aged Finnish men (Kunutsor et al., 2022), while an increased level of ceruloplasmin (CP) is linked to increased HF risk and poor prognosis (Hammadah et al., 2014). It is worth further investigating whether the serum and myocardial copper levels in HF patients maintain a proportional relationship. Experimental investigations have provided evidence that copper chelators can reinstate the expression of critical regulators of mitochondrial biogenesis and enhance cardiac function in rats with HF (Cooper et al., 2004). The influence of copper on mitochondrial function is contingent on its concentration (Isei and Kamunde, 2020). Therefore, copper overload might be a significant factor contributing to mitochondrial dysfunction in HF. However, there is a scarcity of studies examining the connection between copper and HF, and the precise mechanism underlying the relationship between HF and copper overload remains unclear.

Overall, copper ions have been observed in experimental studies to exhibit dual properties in different types of cardiovascular diseases (CVDs). Prospective cohort studies on copper intake in cardiovascular diseases also show inconsistent results, requiring more standardized and larger-scale copper-related therapeutic research on CVD patients. Betaine’s role in HF could represent a new therapeutic approach and might also explain the elevated mitochondrial protein acetylation observed in HF. Copper regulates the production of multiple oxidative centers ROS in the heart mitochondria. Interestingly, when exposed to a copper concentration higher than 5 μM, the production rate of H_2_O_2_ reaches its peak at 25 μM copper, and perhaps copper acts through different mechanisms, including stimulating or inhibiting mitochondrial enzymes, disrupting oxidative centers, dissipating mitochondrial proton gradients, and damaging antioxidant defense systems, depending mainly on the concentration (Isei and Kamunde, 2020).

Metallothionein (MT) is a small protein rich in cysteine that plays a crucial role in binding and sequestering copper as an intracellular copper scavenger. It forms sulfur-copper clusters, effectively sequestering excess copper within cells and minimizing copper toxicity (Thirumoorthy et al., 2011). This function is particularly important as mice lacking the MT1/2 genes display increased sensitivity to copper toxicity (Park et al., 2001). This increased susceptibility leads to severe HF, oxidative stress, and cardiac fibrosis in MT1/2 gene knockout mice, which are further exacerbated by intermittent hypoxia. Conversely, mice with heart-specific overexpression of MT-IIa are protected against intermittent hypoxia-induced cardiomyopathy (Yin et al., 2014). Although genetically induced heart-specific overexpression of MT-IIa in mice does not prevent the development of copper-deficiency-induced cardiac hypertrophy, it does inhibit the progression from cardiac hypertrophy to HF during copper deficiency. This protective effect may be attributed to the attenuation of cardiac lipid peroxidation under conditions of copper deficiency.

Further exploration of the role of MT and copper in disease pathogenesis may lead to the development of targeted therapies to modulate copper levels and mitigate the detrimental effects of copper dysregulation. This could open up new possibilities for treating conditions characterized by copper imbalance and associated complications, including HF and oxidative stress-related disorders. Continued research may also uncover novel mechanisms underlying the protective effects of MT and copper modulation, providing insights into potential therapeutic strategies for these conditions.

#### 4.2.3 Atherosclerosis

At present, atherosclerosis stands as the most common potential instigator of coronary artery disease, carotid artery disease, and peripheral arterial disease (Falk, 2006). Atherosclerosis can lead to the formation of an unstable state, which may result in plaque detachment and damage to the arterial wall (Herrington et al., 2016). It is worth noting that the development of atherosclerosis is associated with the introduction of copper-stable molecules. The buildup and oxidative modification of surplus low-density lipoprotein (LDL) cholesterol are acknowledged as the central processes in the development of atherosclerosis (Ferns et al., 1997). The decrease in ATP7A expression can diminish the cell-mediated oxidation of LDL in THP-1 macrophages (Qin et al., 2010) potentially reducing macrophage infiltration and may reduce macrophage infiltration (Kim et al., 2012). ATP7A, which is the primary efflux enzyme for cellular copper metabolism, is downregulated in response to a decrease in intracellular copper ions, although this could also be a consequence of copper ion accumulation in the serum. Furthermore, both ATOX1 and ATP7A are involved in copper-induced growth of vascular smooth muscle cells (VSMCs), and the migration of these cells is a crucial factor in atherosclerosis and vascular injury (Ashino et al., 2010; Kohno et al., 2013) and the migration of vascular smooth muscle cells is a key factor in atherosclerosis and vascular injury. Additionally, a recent study reported a positive correlation between serum copper levels and the formation of atherosclerotic plaques. Elevated copper levels regulate lipid metabolism, LDL oxidation, and inflammatory responses, thereby elevating the likelihood of developing atherosclerotic heart disease (Kunutsor et al., 2021). Previous research has demonstrated that intracellular copper ions can inhibit the inflammatory pathway, thus reducing the development of atherosclerosis. As an illustration, Zhao and colleagues found that Cu^2+^ coordination polymers had the capacity to suppress the Notch signaling pathway, which plays a pivotal role in regulating chronic inflammation in individuals with atherosclerosis (AS). Consequently, this intervention led to a notable reduction in inflammatory occurrences within the atherosclerotic region (Zhao et al., 2021). Likewise, Wang and co-researchers observed that copper supplements had the ability to hinder the development of atherosclerotic lesions. This was achieved by lowering the rates of endothelial cell death, reducing cholesterol and phospholipid levels in affected tissues, and diminishing the size of atherosclerotic lesions (Wang et al., 2021). However, further investigation is needed to determine the optimal dose of copper supplementation to reduce atherosclerosis since both copper deficiency and excess are associated with an increased susceptibility to this condition. Interestingly, Koksal et al. found that cellular copper levels were higher in pathological inflammatory diseases such as atherosclerosis, which contradicts previous studies (Koksal et al., 2007). The Fenton reaction, one of the most significant metal-mediated reactions, is a key biochemical reaction that leads to oxidative damage (Valko et al., 2005). Copper ions alternate between oxidative and reduced states, generating hydroxyl radicals (Husain and Mahmood, 2019). These radicals engage with DNA and lipids leading to DNA damage and lipid peroxidation. The heightened oxidative stress induced by excessive copper disturbs lipid metabolism, leading to the accumulation of lipids in the intima and the subsequent progression of atherosclerosis (Blades et al., 2021). Moreover, copper can interact with risk factors associated with atherosclerotic processes, such as triggering LDL oxidation and enhancing hydrogen peroxide in conjunction with homocysteine, further promoting atherosclerosis and increasing the risk of cardiovascular disease outcomes (Jain and Mohan, 1991; Gaetke and Chow, 2003; Nakano et al., 2004). However, it is crucial to investigate whether copper accumulation-induced copper sagging exacerbates the inflammatory response of the endothelium and causes endothelial cell damage during atherosclerosis, particularly when oxidative stress is excluded.

#### 4.2.4 Stroke

Copper exacerbates ischemic stroke (Vest et al., 2013). Endothelial progenitor cells (EPCs) are widely recognized for their capacity to stimulate angiogenesis. They have proven to be effective in restoring endothelial function and augmenting angiogenesis in ischemic brain tissue (Peters, 2018). Platelet-1 plays a crucial role as an inhibitor of EPC function (Xie et al., 2010). Jiang et al. conducted a study demonstrating that copper can aggravate ischemic stroke in mice by inhibiting EPC function through an increase in Thrombospondin-1 levels (Jiang et al., 2015). Furthermore, a recent clinical study involving 3,425 subjects aged 20 years and older investigated the relationship between serum copper and stroke risk factors, such as blood lipid level. The study discovered a positive correlation between serum copper and blood lipid levels in females, suggesting that copper may influence stroke risk through its impact on blood lipid levels (Xu et al., 2022). Nevertheless, further confirmation through prospective studies is necessary.

### 4.3 Other ARD

#### 4.3.1 Diabetes mellitus

Diabetes mellitus (DM) is characterized by elevated blood sugar levels, peripheral tissue resistance to insulin, and impaired functioning of pancreatic β-cells. The prolonged presence of high blood glucose levels can lead to extensive vascular harm, impacting organs such as the heart, eyes, kidneys, and nerves, thereby giving rise to a variety of complications (Cho et al., 2018). Among elderly individuals, diabetes remains a significant personal and public health burden, which is a concerning global issue (Sinclair et al., 2020). Elderly individuals with diabetes experience increased mortality rates, as well as reduced functional abilities and quality of life (Bourdel-Marchasson et al., 1998; Gu et al., 1998). Moreover, diabetes in older individuals is associated with an elevated risk of falls, cognitive decline, vascular dementia, and exacerbation of age-related cognitive impairment. Furthermore, diabetes patients are more susceptible to conditions such as hypertension, congestive HF, urinary incontinence, and tuberculosis. Diabetes is indeed a condition that accelerates the development of age-related ailments in older individuals, as referred to as “Isaac’s giants of geriatrics” (Huo et al., 2023). As mentioned earlier, there is a beneficial relationship between advanced glycation end-products (AGEs) and copper deficiency. Although the formation of AGEs is slow in the physiological internal environment, under the high glucose conditions of diabetes, glucose significantly accelerates the modification of proteins, posing a risk factor for multiple diabetes complications. It is important to note that Cu^2+^ with catalytic activity can bind to AGEs and localize in the blood vessels, contributing to the pathogenic damage of atherosclerosis. Additionally, within the context of AD, a positive feedback loop exists: Cu^2+^-catalyzed oxidation reactions are involved in the glycation process leading to the formation of AGEs. Furthermore, copper-binding metalloproteins undergo conformational changes and fragmentation following glycation, ultimately leading to the release of catalytically active Cu^2+^ (Islam et al., 1995; Kang, 2006). However, there have been no reports on the relationship between AGEs and snail disease. In our study, we observed a significant elevation of AGEs concentration in the blood and myocardium of diabetic mice. Our data also demonstrated that AGEs significantly induce myocardial cell death. Importantly, the synergistic toxicity of AGEs and copper is more pronounced, and copper chelators can partially alleviate this toxicity, suggesting that AGEs may promote copper overload-induced cardiac toxicity and contribute to copper deficiency. Further analysis of changes in copper transporter after AGEs treatment revealed that the mRNA and protein levels of the copper import protein SLC31A1 were significantly upregulated, while the output products ATP7A and ATP7B were downregulated. This suggests that AGEs may increase copper accumulation, thereby inducing cuproptosis in cells of diabetic patients. The combination of AGEs and CuCl_2_ also altered protein expression associated with copper cuproptosis and inhibited mitochondrial oxidative respiration. Similarly, in STZ-or db/db-induced diabetic mice, we observed upregulation of heart SLC31A1, changes in copper deficiency-related protein expression, and a decrease in mitochondrial oxidative respiration. To date, only ATP7A protein has been significantly downregulated in vessels isolated from T2DM patients and diabetic mice (Sudhahar et al., 2018), while the changes in other copper transporters ATP7B and SLC31A1 in diabetes remain uncertain. Our results suggest that the upregulation of SLC31A1 in diabetic cardiomyopathy (DCM) may be the cause of copper overload and copper cuproptosisin DCM. The upregulation of SLC31A1 may provide another explanation for the mechanism of DCM (Tsvetkov et al., 2022). Confirmatory animal and human studies have shown that copper levels in the plasma and urine of diabetic patients are higher compared to non-diabetic controls, regardless of the presence or severity of diabetic complications. Elevated copper levels are positively correlated with the production of ROS. Additionally, only one epidemiological study conducted in Japan has reported a positive correlation between dietary copper intake and the risk of type 2 diabetes mellitus (T2DM; Eshak et al., 2018). Furthermore, a correlation has been noted between plasma copper levels and the polymorphism of superoxide dismutase 1 (SOD1) in impaired glucose regulation (IGR) and type 2 diabetes (T2D). SOD1 is an enzyme that relies on copper as a cofactor and has a vital function in the elimination of ROS (Yin et al., 2019). The use of TETA, a highly selective divalent Cu^2+^ chelator, has shown promise in preventing or reversing copper overload in diabetes, thereby inhibiting oxidative stress. Studies involving TETA therapy in diabetic animals and patients have identified and quantified abnormalities in interrelated copper metabolism, characteristic of this systemic copper overload condition (Cooper, 2011). These findings collectively indicate that diabetic patients often experience copper imbalance, oxidative stress, and lipid peroxidation, ultimately contributing to pancreatic dysfunction and exacerbating the disease. Consequently, the downregulation of copper may present a promising avenue for potential therapeutic strategies in diabetes. Nevertheless, additional research is imperative to clarify the impact of copper downregulation inhibitors in animal models of diabetes and to delineate the precise biological consequences of copper dysregulation, both *in vitro* and *in vivo*, particularly in the context of this age-related disease.

#### 4.3.2 Sarcopenia

Sarcopenia is a progressive systemic skeletal muscle disorder that occurs with aging and is characterized by the gradual loss of skeletal muscle mass and function. The onset and progression of sarcopenia involve cell death (Lin et al., 2022) and high levels of pro-inflammatory mediators are considered a diagnostic characteristic of skeletal muscle aging, as chronic inflammation tends to increase with age (Lee et al., 2007). The observed increase in ROS during aging, possibly linked to reduced physical and metabolic activity in older individuals, contributes to the occurrence and persistent resolution of inflammation (Vasilaki and Jackson, 2013). Mitochondria, the primary source of ROS in skeletal muscle cells, generate ROS, particularly superoxide radicals, as a byproduct of oxygen metabolism in the mitochondrial electron transport chain. Additionally, ROS production is also influenced by processes dependent on nicotinamide adenine dinucleotide phosphate (NADPH) oxidase (Sakellariou et al., 2013) and phospholipase A2 (PLA2).

Research has demonstrated that aging animals tend to accumulate ubiquitinated and oxidatively damaged proteins within the myelinated nerve sheaths. This age-related oxidative damage is closely linked to the initiation of pro-inflammatory processes (Opalach et al., 2010). In the context of copper-induced sarcopenia, Cu^2+^-elesclomol has been reported to be transported to mitochondria, where it is reduced to Cu^+^ and triggers ROS-dependent cell apoptosis. While mitochondrial ROS serve as signaling molecules for cell apoptosis, this pathway may not play a crucial role in copper-induced sarcopenia itself (Nagai et al., 2012), Notably, cells relying on mitochondrial respiration are more sensitive to copper ion carriers compared to cells relying on glycolysis. Excessive accumulation of ion carriers or transport proteins results in copper binding to acylated DLAT, which induces abnormal oligomerization of DLAT and the formation of DLAT lesions. Elevated levels of insoluble DLAT can lead to cell protein toxicity stress and subsequent cell death. Skeletal muscle mitochondrial respiration significantly contributes to systemic aerobic capacity, suggesting a possible link between skeletal muscle aging and copper-induced sarcopenia (Bassett Jr. and Howley, 2000). In the future, copper chelators may hold potential therapeutic effects for sarcopenia, but further research is required to validate their effectiveness in its treatment.

#### 4.3.3 Age-related macular degeneration

There exists a certain relationship between copper accumulation and age-related macular degeneration (AMD), which typically involves ocular diseases, especially a condition known as AMD, an age-related eye disease that primarily affects the macula, a critical area on the retina responsible for central vision and fine detail perception. The association between copper accumulation and AMD is primarily linked to dysfunction in the Bruch’s membrane and the retinal pigment epithelium (RPE) in the retina (Harju, 2022). The Bruch’s membrane is a thin layer situated between the retina and the RPE, playing a crucial role in maintaining the normal structure and function of ocular tissues. The RPE, on the other hand, is a cellular layer located beneath the retina tasked with absorbing and metabolizing waste products and excessive copper within the retina. Furthermore, research has indicated a significant increase in copper within the choroid-RPE complex in late-stage AMD cases (Aloysius Dhivya et al., 2020). The accumulation of copper in RPE cells can lead to damage and dysfunction in these cells, which can adversely affect the health of the Bruch’s membrane and the entire macular region. Research involving zebrafish deficient in copper transport proteins has shown that disruptions in copper transport leading to copper accumulation within retinal cells can trigger endoplasmic reticulum stress and retinal cell death. As age and copper accumulation increase, damage and dysfunction in RPE cells may progressively accumulate, thereby increasing the risk of AMD (Wu et al., 2023). Additionally, AMD is also associated with oxidative stress, inflammation, genetic factors, and other ocular diseases. Therefore, copper accumulation is one potential direct influencing factor or may indirectly impact AMD by generating oxidative stress and inflammation. It is important to note that while copper accumulation may be related to AMD, AMD is a complex eye disease, and the specific mechanisms and risk factors are still under research.

## 5 Conclusion and discussion

Cuproptosis in ARD is a fascinating area of research that has garnered attention beyond its initial association with tumors. This form of cell death, linked to the accumulation of copper, is now recognized as a potential contributor to a range of ARD. As the population continues to age, there has been a noticeable increase in the prevalence of neurodegenerative conditions, cardiovascular diseases, and other ARD. Understanding the role of cuproptosis in these diseases has become increasingly crucial. However, despite the insights gained from animal models, a significant gap exists when it comes to direct evidence of cuproptosis in human cells and tissues affected by ARD. This lack of direct evidence highlights a critical area of uncertainty that needs to be addressed. Furthermore, the specific mechanisms by which cuproptosis leads to cellular and tissue degeneration in ARD remain incompletely understood, representing a substantial knowledge gap. Bridging this gap is essential to develop targeted interventions. There is promise in developing therapies targeting cuproptosis in ARD, such as antibodies and drugs.

Looking ahead, in the realm of ARD, several innovative and forward-looking avenues for future research emerge. One such avenue is the potential for personalized medicine in ARD, where understanding individual variations in copper metabolism and susceptibility to cuproptosis could lead to tailored treatments, optimizing therapeutic outcomes. To support this personalized approach, the development and application of advanced imaging techniques, such as high-resolution copper imaging in human tissues, offer the promise of providing invaluable insights into the spatial and temporal dimensions of copper accumulation and its intricate relationship with cuproptosis in ARD. Complementing these advancements, the integration of multi-omics data, including genomics, proteomics, and metabolomics, can provide a holistic understanding of the molecular pathways involved in cuproptosis within ARD, potentially revealing novel therapeutic targets through a systems biology approach. Furthermore, delving into the intricate interplay between cuproptosis and neuroinflammation, especially in neurodegenerative ARD like AD, can shed light on the role of copper dysregulation in perpetuating chronic inflammation, offering novel insights into disease mechanisms. In the quest for early diagnosis and effective disease monitoring, research efforts should focus on identifying reliable biomarkers associated with cuproptosis in ARD, which could significantly impact intervention strategies. Clinical trials designed with precision will be crucial in assessing the efficacy of interventions targeting cuproptosis. These trials have the potential to usher in groundbreaking therapies for ARD, translating research findings into tangible patient benefits. Cross-disciplinary collaborations, uniting experts in copper biology, neuroscience, immunology, and computational biology, are pivotal in fostering innovative approaches to tackle the multifaceted nature of cuproptosis in ARD. In parallel, as therapeutic interventions targeting cuproptosis evolve, addressing ethical and safety concerns related to copper chelation or manipulation is paramount. Future studies should embark on comprehensive explorations of these aspects to ensure the safe translation of research findings into clinical practice. By weaving these forward-looking perspectives into the fabric of future research endeavors concerning cuproptosis in ARD, we aspire to attain a more profound understanding and cultivate more efficacious strategies for prevention, control, and treatment.

## Author contributions

HF: Conceptualization, Funding acquisition, Investigation, Methodology, Resources, Writing – original draft, Writing – review & editing. KW: Funding acquisition, Resources, Writing – review & editing. XZ: Writing – review & editing. BS: Writing – review & editing. TY: Writing – review & editing. TL: Writing – review & editing. GG: Writing – review & editing. WL: Writing – original draft. CL: Writing – review & editing.
